# Online ‘chats’: fostering communitas and psychosocial support for people working across arts and play for health and wellbeing

**DOI:** 10.3389/fpsyg.2023.1198635

**Published:** 2023-07-24

**Authors:** Katey Warran, Laura H. V. Wright

**Affiliations:** ^1^WHO Collaborating Centre for Arts and Health, Social Biobehavioural Research Group, Department of Behavioural Science and Health, Institute of Epidemiology and Health Care, University College London, London, United Kingdom; ^2^Edinburgh Centre for Research on the Experience of Dementia, School of Health in Social Science, University of Edinburgh, Edinburgh, United Kingdom; ^3^Children and Young People Thematic Hub, Moray House School of Education and Sport, University of Edinburgh, Edinburgh, United Kingdom

**Keywords:** community, arts and health, play, qualitative, online interaction, COVID-19, psychosocial support, communitas

## Abstract

Loss of work, furlough, and increased social isolation were prevalent for many working in the broad context of cultural and community engagement for health and wellbeing. This study set out to explore if and how regular online group interactions may foster social cohesion and provide support for these individuals during the critical time of the COVID-19 global pandemic. It was conducted in the context of the ‘social cohesion chat’ series led by a network called the Arts Play Health Community which was initiated in response to the pandemic as a way to bring those working in or connected to arts, play and health together during times of social isolation. Two qualitative focus groups with creative, participatory components were conducted with artists, researchers, evaluators, and arts/play managers (*n* = 11), and then analyzed using thematic analysis. Researcher ethnographic reflections and fieldnotes were also collected and analyzed. The authors engaged in reflexive online discussions to integrate and synthesize findings across different data. Four themes were constructed through the analysis procedure: (1) ‘Building an online community as processes of communitas’, spotlighting the importance of the non-hierarchical structure of the ‘chats’ particularly in relation to there being ‘no end goal’ to the online dialogues; (2) ‘Individual and shared emotional experiences’ that underpinned feelings of connection to others and the online space; (3) ‘Psychosocial benefits’ such as improving confidence and providing an opportunity to ‘have a voice’ in the community; and (4) ‘The importance of facilitation’, highlighting the opportunities the chats provided for participants to feel validated and valued as an active member of the community. The article concludes that constructing an inclusive and welcoming online community, where active participation is at the heart of regular social interactions can provide support for those working across arts and play for health and wellbeing. This was particularly important during the societal turbulence of the COVID-19 pandemic. It further concludes by noting the unique structure of these online dialogues as not being connected to institutions, with this playing a key role in allowing those in the community to ‘be themselves’ within it.

## Introduction

There has been increasing interest over the last decade in the role of community and cultural engagement in improving and sustaining health and wellbeing (Fancourt and Finn, 2019; Fancourt et al., 2021). This includes a range of creative, arts, cultural and play-based group activities and experiences, including performing arts, free play, visual arts and crafts, and museum attendance, to just name a few examples. Whilst there is a large literature on the relationship between these activities and their impact on health and wellbeing (Munford et al., 2017, 2020; Fancourt and Finn, 2019), there’s very little literature that has explored the working lives of those operating within this landscape. The few studies that do exist have examined the detrimental impact on health of working as a professional artist, particularly in the context of the music industry (Ascenso et al., 2016; Gross and Musgrave, 2020; Musgrave, 2022), with less focus on those specifically working within the community and health sectors. As an intersectoral space, this includes many more careers beyond artistic practice, including administrators and managers working for arts and cultural organizations, in the third sector and for community organizations, researchers, students, and evaluators, many of whom are freelance, have portfolio careers, or are on short-term contracts. The nature of these careers therefore tends to be precarious.

Within research on creative and cultural careers specifically, and indeed in broader studies of psychology and happiness at work, it has been suggested that social capital, social relationships, and social networking are important to sustaining meaningful careers and to securing work opportunities, particularly in the context of precarity (Gerber, 2017; Fox et al., 2021). Literature in social psychology also highlights a strong rationale for forming meaningful communities and social groups to attain important psychological resources that are important to health and wellbeing (Jetten et al., 2011; Haslam et al., 2018). The increasing literature on the social determinants of health shows strong rationale that meaningful social relationships at work are important not just to career progression and to our social lives in the workplace, but to our mental health, wellbeing and quality of life too (Hori et al., 2019; Fox et al., 2021).

However, when the COVID-19 pandemic began and after the onset of the first UK lockdown in 2020, social lives were disrupted in unprecedented ways, with many workplaces needing to find new ways to operate at a distance, such as using digital technologies. These changes disrupted working lives leading to major psychosocial impacts, particularly for those with freelance or precarious careers, with research showing that those working within the cultural industries were particularly adversely affected (Spiro et al., 2021; May et al., 2022; Warran et al., 2022). It has been suggested that this was, in part, due to pre-existing structural challenges, such as in relation to the complex economic structure of these industries where many workers move in and out of contract work, take on unpaid work, have to manage periods of unemployment and/or work in other in other industries in order to secure income (Throsby and Zednik, 2011; Gerber, 2017; Smith and Thwaites, 2018). Within the broader context of the pandemic and on a global level, people increased virtual interactions across both their professional and personal lives and sought new ways to connect during times of isolation (e.g., use of Zoom, Facebook Video, and Microsoft Teams). This included online choir groups, reading clubs, games nights, educational classes, and social groups, some of which were groups that used to meet in-person that were adapted for online forms, and others were new activities that formed in response to the need to connect during times of mandated social restrictions (Bradbury et al., 2021; Mak et al., 2021; Warran et al., 2022).

However, very little research has explored specifically what makes an *online* group interaction supportive for social connections, health and wellbeing. There are a few exceptions, for example weekly online music appreciation sessions for those who use Cochlear Implants in the US have been found to support wellbeing and self-care (Kovach et al., 2022) and weekly singing groups for people with chronic obstructive pulmonary disease have shown possible improvements to depression (Philip et al., 2020). Yet, these groups have focused on particular health conditions and have not explored the relationship between social connections and wellbeing in the context of working lives. One further example is a study exploring an online activity with the aim of helping people to maintain social connectedness when face-to-face interaction was not possible during COVID-19 (Groups 2 Connect), which found that short 15-min structured (e.g., including goal setting) online social engagements could increase perceived ability to stay connected and well-being (Bentley et al., 2022). Although exciting findings as this study highlights the potential for digital interactions to meet social needs, this was a short-term intervention recruited from ‘the general community.’ Thus, it was not targeted to meet the needs of specific groups of people (e.g., those with precarious careers), nor able to reveal what may make a thriving longer-term ‘community’ online. This is important as research exploring ‘offline’ social groups has shown that benefits of group engagement may increase with longer-term commitments; for example, music groups have shown that psychological improvements are stronger in the period between 6 and 12 weeks than 0–6 weeks (Fancourt et al., 2019a,b), and that group identities form after 8 weekly (90 min) sessions of singing and song-writing combined with socializing (Dingle et al., 2020). In addition, it has been shown that cultivating a ‘sense of belonging’ to a community over time may be supportive of health and wellbeing (Jetten et al., 2012; Haslam et al., 2018).

In sum, there is a clear need to provide support for those working in precarious careers during the pandemic, such as those working in the various careers connected to cultural and community engagement for health and wellbeing that have been overlooked in previous studies. The research highlights that it is possible that social groups may be able to provide social support, but less is known about how to create an online sustainable ‘community’ that is supportive. In view of this, the aims of this study were to explore:

1. Whether it is possible to build a meaningful online community for those working in the broad context of cultural and community engagement for health and wellbeing; and.
2. If and how such a community may provide psychosocial support for those who engage with it.

## Materials and methods

### Context: the arts play health community

In the wake of the onset of the first UK lockdown due to the COVID-19 pandemic in 2020, the authors (KW and LW), experiencing their own uncertainties and a desire to support social cohesion and connection, set up a new network called the ‘Arts Play Health Community’ with an associated weekly online initiative called the ‘Social Cohesion in Social Isolation Chats’ via Zoom. The first of these ‘chats’ took place on Wednesday 18 March, just days before the first lockdown came into place on 23 March 2020. The community had two aims: (1) to bring together those working within and across cultural and community engagement for health and wellbeing, with a particular emphasis on ‘arts’ and ‘play’; and (2) to provide an inclusive online space for dialogue to provide support during times of social isolation. In relation to the first aim, this was the result of the recognition that arts and play have close connections in how they intersect with health experiences, but with no networks set up to encourage knowledge-exchange between these fields. We welcomed broad understandings, experiences, and conceptualizations of ‘art’ and ‘play,’ with those engaging self-identifying with these terms. Within our own conceptualization of the community, we drew upon, yet were not confined by, definitions of ‘the arts’ as outlined in the World Health Organization scoping review on arts and health (Fancourt and Finn, 2019), ranging from performing arts activities, visual arts participation and digital arts to literary, cultural and engagement, also recognizing the conceptual complexities of defining these multi-modal activities, and of ‘play’ from the United Nations General Comment 17 on the right to play. We valued the diverse and complex ways of conceptualizing play across disciplines and sectors, and sought to move away from definitions, respecting the critique of universal play definitions that are often premised on developmental models based on middle-class children (or adults) of the minority world (Goncu et al., 1999; Gaskins and Miller, 2009). The authors were also already in the process of organising an international conference on arts and play for health and wellbeing ahead of the pandemic, and the new community built on the foundations and connections that were already in development.

The ‘chat’ series was advertised to academics, evaluators, health practitioners, artists, arts/play managers or administrators, and community members such as those with lived experience. The structure was a weekly discussion taking place 4–5 pm (GMT/BST) that centered on a ‘hot topic’ or ‘critical question’ related to arts, play and health and was facilitated by either the co-founders (KW and LW) or a guest facilitator (who was often someone who had already participated in previous chats). Each week, the authors of this article would facilitate introductions, often using a creative prompt, and then hand over to the guest facilitator to lead an open discussion with around 4–5 key questions (as prompts for dialogue) that they would like to discuss with the group. Everyone who attended was encouraged to actively engage (either verbally or through use of the Zoom chat function) and the priority was to ensure that all those attending felt welcome to contribute. ‘Presentations’ were not encouraged, the sessions were never recorded to create an environment where everyone was comfortable to speak, and the only ‘aim’ of the ‘chats’ was to engage in a connective dialogue. The first chat had 25 participants, including individuals from Scotland, Ireland, England, Benin, Canada, Cyprus, Spain, and other locations, spanning diverse sectors including those from academia, the non-profit sector, and public health, as well as freelancers, artists and policy makers. Following this, the group maintained around 8–12 participants per week. In October 2020, the chat series went bi-weekly, and then in July 2021 it went monthly, in response to the changing context of the pandemic. The Community’s online conference took place 15th-18th February 2021 for over 200 delegates, and an online Slack forum was created for discussions across the conference and chats which is still in use. At the time of writing, the Community has 378 people on the mailing list and approximately 4–10 people attend the monthly chats. The data collection for this research took place between March and October 2020.

### Methodology

A key focus of this study was to explore if and how meaningful ‘social’ experiences form through relational engagement online (e.g., through dialogues, creative prompts and facilitation) during times of precarity, and how these experiences may connect to perceived sense of belonging to an online ‘community’. Thus, it was important to understand group dynamics and how individuals feel within an online group space, as well as view them in light of the context of the global pandemic. We therefore employed a qualitative approach to take into consideration the meaning individuals or groups construct within a social world, placing importance on interpreting complexity (Creswell and Creswell, 2013). In particular, we used focus group methodology as a way to explore the ‘negotiation of meanings through intra- and inter- personal debates’ allowing access to a range of communication forms such as ‘joking, arguing, teasing and recapturing past events’ and enabling an understanding of group dynamics (Crang and Cook, 2007; Liamputtong, 2015). Researcher observation of these interactions in the focus groups were important to explore how the group negotiated their experiences in and through dialogue with others. In the groups, we also drew on participatory, arts-based approaches to create space for participants to engage in reflective processes and for open dialogue to emerge (see Methods). Recognizing that participant experiences were constructed across time and that our own engagements in the community played a role in the construction of it, we also drew on inspiration from ethnographic approaches to explore the ‘social world’ of the chats through our own reflexive processes.

### Ethics statement

The study adhered to ethical research guidelines and principles for safety, dignity, rights, and well-being of the participants (Morrow, 2009). Our ethical procedure and practice addressed critical elements of voluntary and informed ongoing consent and assent, limited confidentiality, anonymity, do no harm protocol, power imbalances between researchers and participants, and use of data. All participants read an information sheet and provided written informed consent to participate. While not ‘high risk,’ the limits of confidentiality were shared with participants during the consent process so that all were aware that if anything was shared that put the individual or others at serious risk to themselves or others, and/or if incidents of abuse were witnessed, reported, or suspected, the research team would need to report it to someone who could provide support (i.e., appropriate authorities). The ethics in this research thus adhered to procedural ethics and integrated an ‘ethics of care’ that respects and recognizes participants as relational, and further emphasized the value of participants being respected, treated with dignity, and listened to in the research (Bussu et al., 2020). The participants themselves were voluntarily part of the Arts Play Health Community that regularly connected in online dialogue spaces and thus also practiced an ethics of care to one another during chats and focus group discussions. Pseudonyms are used throughout the reporting of findings to preserve the anonymity of participants.

### Participants

Participants were recruited for the focus groups during August 2020 by sharing information about it verbally at several online chats and emailing those who had attended the online chat series. Eleven adult participants in total took part in a focus group during September 2020: 6 participants in the first focus group and 5 in the second. This included 10 female participants and 1 male participant from the United Kingdom. Table 1 shows the job roles of those who participated. The kinds of contracts participants had have been noted to highlight the precarity of the job roles that they had at the time of joining the focus groups, with many either on short-term contracts or working freelance. It should be noted that, at the time of delivering the focus groups, the authors had precarious job situations too: both in the final year of their doctoral studies and undertaking part-time research/teaching alongside freelance consultancies to supplement their funding.

**Table 1:** Job roles of those who participated in a focus group.

Primary role	*
Arts manager – contracted	4
Artist – freelance	2
Researcher - contracted university	2
Researcher/evaluator - contracted third sector	1
Researcher/evaluator – freelance	1
Arts manager – freelance	1
Researcher - student	1

*11 people participated, with one person occupying two of the categories listed.

### Methods

Focus groups were facilitated by the authors (KW and LW), lasted 90 min and included drawing on a participatory creative method known as the ‘river journey.’ The river journey originated from a Most Significant Change participatory rural development monitoring and evaluation tool (Willetts and Crawford, 2007) and has been modified in different forms to be used as a reflective participatory tool. The ‘river journey’ has been used globally in multiple research studies with children, young people, and adults (Lee et al., 2020; Wright, 2021; Finn et al., 2023; Wright et al., in preparation). The tool was selected for this study as it aids participants to reflect on an experience over time, and aligned with the arts-based and dialogic experience of the social cohesion chats that the research participants were familiar with.

The river journey process involved inviting participants to reflect on their experiences of engaging in the chats from the start, to the middle, and to present and future. Participants were asked to draw a river across a piece of paper with start, middle, and future written across the page. They were invited to draw images and use text to reflect on strengths and challenges of their journey as participants and facilitators in the chats. They were then welcomed to share their rivers (optional), each taking turns in the online circle to describe what they had drawn and why. After sharing, a focus group discussion was facilitated using semi-structured questions to probe further on key areas of the stories (see Supplementary Appendix). Extensive notes of the discussions (including writing down participant quotes verbatim) were taken by the researchers and the discussions were recorded and auto-transcribed using Zoom. While the tool proved to be an effective approach with the participants to reflect and engage in dialogue, it could have potential limitations in other studies where participants have less familiarity with one another, whereby participants may feel uncomfortable with sharing personal reflections. In such circumstances, participants are also welcome to write down notes and/or the researcher could host follow up one-to-one interviews.

In addition to the focus groups, the researchers documented notes and reflections from each of the weekly and bi-weekly meetings from March to October, saving reflections in a shared folder. These notes consisted of key highlights and ‘ah-ha’ moments (i.e., pivotal moments that inspired new thought or emotions and/or that stood out to the researchers) from the chats that related to the aims of this research, with pertinent quotes also anonymously recorded. We sought to purposively ‘filter out those elements of the perceptual world that [were] not central to concern in a given moment, and... “filter in” those elements that [were]’ (Schwartz-Shea and Yanow, 2012, p. 79), focusing on key elements of interaction and engagement that related to our study aims. These fieldnotes aided us in capturing ‘rich descriptions of the study context, encounter’ and research activities (Phillippi and Lauderdale, 2018, p. 382) and any situations that disrupted our frame of thinking (Emerson et al., 2011). The fieldnotes were also further supplemented by the researchers own autoethnographic experience in facilitating and hosting the dialogue spaces, including observing and being a part of the dialogue processes. These personal reflections were discussed between the authors through a series of meetings as a form of shared reflexivity. This involved reflecting on our positionality and lived experiences, what we brought to the data and our roles in the cocreation and sustainability of the community. We acknowledged our position as white, middle-class females, who were both active in art (KW) and play (LW) professional spaces and who personally experience enhanced wellbeing through our relationships with art and play. We critically reflected on our situated knowledges and being ever mindful of them as we carried out the study. For example, we discussed our roles as doctoral students in 2020 (which is both a privileged and yet somewhat precarious job situation) and facilitators of some of the chats, such as in relation to the discussions that had most interested us and why, as well as our own experiences and responses to the pandemic on both a personal and professional level. We sought to be honest about affordances and limitations of reflexivity and to hold ourselves accountable to positionality and its effects on our study and participants (Absolon and Willett, 2005).

To analyze our data, we used a reflexive thematic analytic approach to systematically analyze our qualitative data (fieldnotes and focus group transcripts), drawing on Daly et al. (1997) and Braun and Clarke (2006, 2012). This involved familiarization with the data and then line-by-line coding, before integration of codes to construct themes that were important to the description of the phenomenon, also exploring patterns in the data (Daly et al., 1997). This was an iterative process of reading and re-reading the data engaging in art and playful process (e.g., listening to music, drawing ideas) while exploring emerging themes (Rice and Ezzy, 1999). We sought to find patterns ‘within and across data in relation to participants’ lived experience, views and perspectives’ (Clarke and Braun, 2017, p. 297). For the focus groups, the authors independently analyzed and created themes from the transcripts and written notes from the discussions using both Nvivo and hand coding. As this was a small-scale study, each author engaged in a process that was familiar to them, facilitating immersion in the data and co-analysis processes. The authors came together in a series of meetings to discuss the findings and collectively co-construct the final themes.

## Results and discussion

The following section explores four interconnected themes that were constructed through the analysis procedure: (1) Building an online community – processes of communitas; (2) Individual and shared emotional experiences; (3) Psychosocial benefits; and (4) The importance of facilitation. Underpinned by our qualitative interpretative processes, we acknowledge that our findings were ‘brought into existence through the framing of [our] research questions’ (Schwartz-Shea and Yanow, 2012, p. 79), and that we drew upon wider reading and theories to interpret and ‘work with the data’ (Gerber, 2017, p. 142). As such, we present our findings alongside the wider literature that we both used to interpret our data and to position it within following co-construction of our themes. Together, the themes and analysis explore how a meaningful community was fostered for participants who engaged in the chats that provided psychosocial support during the uncertain context of the COVID-19 pandemic.

### Theme 1: building an online community – processes of communitas

Our chats supported with developing a “sense of belonging” (Mina) for participants, with this belonging tied to postmodern theorizations of ‘liminal communities’ and ‘networking’. Whilst liminal communities may be considered temporary in nature (Delanty, 2010), we found that there was a kind of liminality to our community that provided a sustainable community that held meaning for those who engaged. This was a liminality that was connected to the transient nature of people moving in and out of a ‘virtual’ space that was both consistent (i.e., there to engage with for the chats) whilst also impermanent (i.e., lasted only for the time that the chats took place). A key part of developing sustainability within this liminal landscape was the opportunity for active engagement which was described as creating “a sense of being part of a community” (Sally). This active participation was viewed in contrast to other online activities, such as work meetings and presentations. At these more passive events, participants said that they would “just start doing work” or “drift off” (Marie-anne); whereas the chats provided a space to be more actively involved:

*“…there’s a level of engagement, which I think is maybe about being able to chat alongside the conversation, giving everybody that space and permission. So similar to [other participant], I felt like I was allowed to be there and I allowed myself to use the time to reflect and to think about my practice and think about my knowledge, think about my networks. And I found that there were opportunities and sometimes the people that I met kind of sparked an idea. Sometimes it’s the opportunity to facilitate or put together blogs and sometimes it’s just, like, the sort of developing a sense of community” (Marie-anne).*

This active participation was also viewed as a kind of “giving to the group” in contrast to the feeling of being “lost” from “being furloughed” (Naomi) and also fostered a “safe space to share” (Mina). Nonetheless, participants also felt that there was an important balance of active participation and reflection, with one participant describing this as like “sitting on a riverbank and watching the river go by” because “it’s a contemplative space as well [as a participatory one]” (Mina). This opportunity to choose to either participate or reflect was important.

As has been argued by Jamjoom (2022), the pandemic prompted ‘a continuous liminal state’ where we were ‘always transitioning’ and ‘searching for new meaning’ (p. 1316). Our participants experienced what Turner (1975) has described as liminality *together* – a sharing of this flux – developing into a co-construction of *communitas* (an unstructured state [‘antistructure’] that contrasts a structured community). Continuity can be a dimension of communitas (Turner, 1975, p. 13) and the bonds are ‘undifferentiated, equalitarian, direct, nonrational, I-Thou or Essential We relationships, in Martín Buber’s sense’ (Turner, 1975, p. 47). Individuals who engage exist as separate, autonomous people, but they connect on a very ‘human’ level in a relational way that unites everyone in a sense of shared experience. The chats provided such a context in which participants came ‘as individuals’ where they engaged in an ‘unstructured’ process that united them in a sense of community. This *communitas* was ‘held’ in the virtual space and available for individuals to engage with in the ways in which they chose to in amongst the broader shifting social context of the pandemic.

Moreover, the dialogic nature of our chats as a “sharing space” where there was “no end goal” (Sally) and a lack of institutional hierarchy (i.e., everyone had different institutional affiliations and there was no formal organization behind the running of the chats) enabled participants to engage as what Turner (1975) describes as ‘authentic individuals’ (p. 54). They felt ‘liberated’ from the formal social structures of their own workplaces (“refreshing contrast,” Sally) and navigated their own relationships between their personal and professional identities in a group setting with others sharing similar challenges and opportunities. When exploring what participants wanted from the Arts Play Health Community, several participants reinforced the importance of sharing, seeking a “space to share personal reflections and own experience” and to “connect on collective ideas” (Field Notes, 01/04/20). Furthermore, participants felt the chats acted as “sanctuary spaces where people find their refuge” (Field Notes, 01/04/20).

Accordingly, whilst the group involved active participation as a key part of feeling part of the community, the dynamic and liminal nature of the group was important: it was constantly changing and had a ‘drop-in style.’ One participant felt that the balance of having a consistent group, alongside inviting new people was important, but also questioned how to keep that kind of fluidity going:

*“If we just have new people all the time it would not work. And if we have the same people all the time, it does not work. So how do we keep that dynamic thing that does not create a bigger group or too small a group?” (Marie-anne).*

In addition, participants felt that the drop-in nature of the group was important to ensure that participation was ‘manageable’ (Marie-anne) and wasn’t too demanding of time and personal resources:

*“The timing and the range of ways to engage and being able to come late and to drop in and out and still get something valuable out of it. I found that this was a relatively accessible format for me.” (Marie-anne).*

*“I think it is really important that you feel that you can come if you want to. But if you do not, it’s not like you have missed out on something or you are going to need to catch up before the next session…. You do not feel that you have to come to every session, but the invitation is there if you want to.” (Sally).*

This lack of structure could be viewed as a risk to the group falling apart. It has been suggested that digital forms of connection are less connective than in-person modes and that we are in an age of increasing isolation, despite more opportunities to ‘connect’ (online) than ever (Collins, 2020). Whilst we did see fluctuating numbers of people attending the chats and, over time, the ‘core’ of the group has changed as some people have engaged less, with others taking more of an active role, we have found that the unstructured nature of the group has provided an online ‘space’ that people can choose when and how to engage in a way that suits them. The people come, go and change, but the space is a consistent support that is ‘always there’. In this sense, our chats align with Rainie and Wellman (2012)‘s idea of ‘networked individualism’ whereby digital networks offer liberation from the ‘restrictions of tightly knit groups’. One can choose how to engage in different social settings and have multiple forms of belonging that allow for self-development and decision-making, also empowering individuals. Our chats supported with breaking down hierarchical conceptions of networking (e.g., networking in order to ‘make it’ or further careers; Hesmondhalgh and Baker, 2011), drawing on a more liberated conception driven by passionate individuals engaging in shared experiences during the fluctuating, and uncertain, context of the pandemic.

Nonetheless, several participants did sometimes feel guilty for not coming. One said that “you feel bad” when you are “not able to attend so many” (Charles), another that they had “a little guilt box” (Naomi) and another that they “battled” with guilt when they felt “tired” or “did not have the time for that [to attend]” (Sally). Yet, this also suggests that despite there being no formal ‘need’ to attend, participants felt enough commitment to the group that they felt they ‘should’ be there, thereby implying that the group held meaning for them.

Another reason participants’ felt able to drop in and out of the chats was that they were perceived as fostering a feeling of inclusivity and diversity (“there were new people all the time, new topics and people came for range of backgrounds,” Marie-anne). This is interesting because it contrasts theories that suggest ‘barriers to outsiders’ are needed in order to create meaningful group solidarity and identity (e.g., Social Identity Theory, Tajfel and Turner, 2004; Interaction Ritual Chains, Collins, 2004). The fact that ‘anyone’ could join was part of the group’s identity. One participant described the facilitation of the group as “incredibly kind of accessible” (Naomi) and another participant wrote “accessible” on their river journey (Marie-anne). Another participant also felt that the chats supported with reflecting on what inclusivity is at large. They noted that the chats supported them to “rethink what my voice is and rethink the voice of those that are marginalized,” with the “social cohesion space, a bit like a horizon that does not have a middle and an end” (Mina), allowing time for reflexivity.

The diversity of the group was also important to participants, bringing together people from different backgrounds and with different perspectives:

*“And but also lots of people I’ve never met before. Which kind of leads up to here to the, sort of, the different perspectives and how that brings connection.” (Sally).*

A key element of these feelings of inclusivity and diversity were also the ways in which the online space felt “supportive and safe” (Marie-anne) and was “a safe space to come” (Mina), which enabled it to feel inclusive for those who engaged. However, one participant also stated that they acknowledged an online format is not “accessible for a lot of people” and that even during the focus group they were experiencing “problems because my internet is unstable” (Marie-anne). Further, we have only been able to reflect on the experiences of those who came to the chats, rather than those who did not come (who could feel differently about the accessibility of the group). There are clear ways in which the chats could be viewed as not accessible (e.g., digital poverty) but, for our participants, the ‘open’ nature of the group supported with creating *communitas* in a way that was meaningful and supported self-development.

### Theme 2: individual and shared emotional experiences

The data highlights both an individual and collective understanding of emotions. Drawing on Ahmed (2014) and Burkitt (2014), we recognize a relational conceptualization of emotions (i.e., as constructed through relations with others) and seek to draw on wider social processes to explain the role of context in the construction of shared emotional experience. This also connects to Collins (2004) ‘emotional-entrainment model’, which draws upon Goffman (1967) and Durkheim (1995) to argue that social solidarity is created through ‘interaction rituals’ whereby emotional energy in individuals (created through shared mood and attention) binds people together across social interactions. In a similar way, our data showed that shared emotional experiences in the online space fostered a sense of belonging to a community.

The participants involved in the study were active in chats that began during the start of the global COVID-19 pandemic. Most participants were unfamiliar with online meetings and the Zoom platform, as such, engaging in an online community was a new experience where many emotions and reflections were emerging. One participant spoke of our experience as an “element of collective emotion” (Poppy) and “a shared feeling/sense of hope” (Field Notes 15/04/20). Thus, the online space provided a platform for both verbal and embodied expressions and sharing of emotion.

During the river journey process several participants also expressed feeling apprehensive at the beginning of joining the chats and then over time felt it was a positive experience. Words such as “belonging,” “supportive,” “safe,” “welcoming,” “connected,” and “respected” were written on the journeys and voiced in the discussion. Participants also expressed feeling thankful at the end of the focus groups, expressing gratitude for their experiences in the chats. As people described their emotions on their rivers, others in the group either verbally or non-verbally (i.e., through nodding) confirmed they had experienced such emotions, suggesting the emotional underpinnings of the community were grounded in similar experiences.

As previously explored, some participants spoke of feeling “guilty” for not being able to attend, yet also grateful for being welcomed back each time. For some, the dialogue spaces were out of their regular comfort zone and yet a valuable space for collective reflections and emotions to be shared on their own practice and personal lives during uncertain and changing times. Importantly, these emotions were described as fostering a sense of “working family” (Ava) in the context of their changing working lives and previous relationships in their working networks. In this sense, their engagements in the chats created emotional-entrainment akin to Collins (2004) conceptualization that fostered a group solidarity underpinned by shared precarity and the need for meaningful working relationships:

*“This became my working family. That was so important and hard to articulate.” (Ava).*

*“Even if you do not show up you are still invited. No ask to qualify you to still be invited… it’s a Family-esque thing. You will keep being asked even if you cannot make it or you are too tired. Sometimes if there are other things, or you feel guilty, you will still be asked.” (Ava).*

Although online, the facilitators created space for participants to connect with their own bodies and in tactile processes with materials in their own homes. For example, in one session the group closed with an “armchair” choreography where each participant chose two simple movements to express while the facilitator played an instrument (Field Notes 06/05/20). On another occasion participants were invited to create an image that meant ‘connection’ to them (Field Notes 20/05/23), and in other chats participants were welcomed to share an object or an experience from the week that reflected their wellbeing. These experiences fostered a unique form of intimacy connecting participants with further insights of one another’s personal lives and their connected lives through embodied expression and sharing. As such, experiences of the chats did not exist entirely ‘online’: there were many ways in which engagements ‘spilled out beyond’ (Fawns et al., 2020, p. 2) the virtual, uniting physical and digital spaces in ways where the lines between the two could not be disentangled, and whereby work and personal merged. It was the coexistence of embodied emotional experiences and shared online emotional experiences fostered through relational experiences that enabled the participants to feel part of the group.

### Theme 3: psychosocial benefits

Interconnected to these emotional experiences, the data showed psychosocial benefits for participants through engagement in the chats. Psychological research highlights that social relationships are pivotal for psychological wellbeing (Haslam et al., 2018). While much research shows the critical role of in-person face-to-face relationships, Waytz and Gray (2018) posit that online technologies may be able to ‘facilitate humans’ connectedness by complimenting existing offline relationships and allowing social connections when they would otherwise be unachievable’ (Marinucci et al., 2022). This is particularly pertinent in the context of the pandemic, where online connection acted as protection for harmful impacts of isolation, particularly for those with less opportunities for in-person interactions (Marinucci et al., 2022). This was relevant to the participants of this study whose careers were heavily impacted by mandated restrictions during the pandemic, such as sites of artistic and community engagement closing and less opportunities for in-person networking, thereby resulting in primarily work-from-home lifestyles for a sustained period. The chats created an online space for social connection that provided psychological resources for our participants during the uncertain time of the pandemic. Further, what was interesting in our study was that participants remarked finding the online chats a space that continued to hold value, even when restrictions eased and people were able to meet more regularly in person. This is further supported by the continuing attendance at the online chats beyond the period of data collection for this study.

Of note, self-confidence was expressed as an important mechanism underpinning beneficial changes to psychosocial wellbeing. This was created and sustained through a relationship to oneself, others in the group, and the larger personal and professional networks of our participants. This self-confidence was connected to participants having the space to express themselves through dialogue without being confined by structures (i.e., the unstructured communitas) and also, on a few occasions, through embodied mediums (e.g., collective poem writing, drawing to start). This psychosocial resource of self-confidence also acted as a driver for people wanting to return to the chats, as participants felt energized and motivated to attend. For example, one participant shared feeling “a lot more confident and [that she] started to look forward to the sessions which was positive for me” (Maya). This participant looked forward to the chats because they were confident in attending them and confident that they would have a positive impact. This reinforces Collins (2004) theory that the socioemotional benefits received through meaningful group interactions also leave participants wanting to go through that experience again in order to increase benefits further. Additionally, this self-confidence was further reinforced in and through relations with others in the group. Participants reflected on their own skills (e.g., of facilitation, see Theme 4) which were positively reinforced by others (e.g., through nodding at the focus group, positive feedback at the end of chats given to one another), further enhancing sense of self-confidence for participants both during and beyond the chats, such as part of their own professional practice (e.g., in work activities).

A key component of the psychosocial wellbeing experienced through the chats was also ‘being heard’. This enhanced feelings of self-worth, respect and confidence for the participants, particularly those who felt undervalued within their own workplace of freelance roles:

*“Just before lockdown I went from a salary employed role, to being freelance. That move from not having a job title that said ‘who I was’ to being invited and valued ‘as myself’ rather than as a representative of an organization. Something in that part of the world. You’re welcome and you are valued. Come share who you are, not to speak on behalf of an organization.” (Ava).*

*“In my organization, I am very cautious I am the bottom rung of the ladder, I do basic admin. Encouragement of any kind of form… there is not that space in work. And so, I agree with [other participant], it is something about having that extra oomph of value and peer support and encouragement. A safe space to talk about those things*.” *(Freya).*

The quotes above show the impact on self-confidence and the value of being listened to and heard for participants. Participants who perceived themselves “at the bottom rung of the ladder” in their own professional space found the non-hierarchical and relational format of the chats enabled confidence and new ways of engaging with themselves and peers. This also provided new motivation in both personal and professional lives (“it helped with my motivation,” Maya). This further supported with creating an identity that existed beyond a solely professional identity connected to their formal roles and existing authentically ‘as me’ in the online space.

Another key finding was improved coping, whereby participants felt equipped with new tools that they utilized both within and beyond the chats. This was referenced as connected to the creative ways of engaging in the chats (e.g., through engaging with arts-based activities) and through drawing upon the socioemotional resources gained through engagement:

*“And the coping mechanisms. Also, that’s another little phrase I’ve written down [on the river]. So, my coping mechanisms it really helped.” (Maya).*

Finally, participants stated appreciation for and permission to take *time* for themselves (“It felt so good to have taken that time for me. Which is here a space for me” [Sally]). While the time for oneself was highlighted in the context of attending the chats, this time for self also appeared to have ripple effects on impacting participants’ external professional, personal and relational experiences. Conversations that took place and relationships that formed “kind of sparked an idea” (Marie-anne) and rippled outside of the social dialogue space (e.g., members reached out to one another to share work opportunities and events).

Looking to the wider literature, it has been argued that an entrepreneurial attitude characterized by self-initiative and self-improvement may be important to thrive within the context of neoliberalism and a precarious creative career (including physical and mental stressors), with this need growing as a result of the increasing precarity brought about during COVID-19 (Foucault, 2008; Scharff, 2017; Warran et al., 2022). This presents a case for why psychosocial support may be so essential for our participants, whereby resources such as coping and confidence may contribute to resilience during increasing precarity. There are of course major challenges with addressing structural problems at an individual level, but our study suggests that in an environment of collective and macro-uncertainty (the pandemic) which could not be ‘changed’ an individual level, psychosocial resources are one important form of support.

### Theme 4: the importance of facilitation

Interrelated to all of the themes explored thus far, this final theme examines the importance of the role of being a facilitator of a chat. This role meant choosing a theme and facilitating the dialogue of a session and was usually someone who had previously engaged in the chats. This played a pivotal role in fostering the unstructured hierarchy of the space (i.e., anyone could ‘lead’), thereby contributing to processes of communitas, as well as providing an opportunity for more meaningful relational experiences. The role further supported with personal and professional development too, such as in relation to building confidence, as previously explored (“helped with my confidence as well my self-belief,” Maya) and feeling validated:

*“That moment when you asked me to facilitate. I felt validation and value which meant a lot to me… It (facilitating) really helped because it helped validate my thoughts” (Maya).*

This validation was also viewed in professional terms, with one participant commenting that they felt their “recent work” (research) was not “relevant” anymore but coming to the chats and facilitating on their work helped to “acknowledge” their efforts (Mina). These experiences connected to participants’ emotions too, such as feeling valued, “quite honored” (Naomi), and providing a sense of purpose. This purpose was important from a professional perspective, for example one participant commented that they were feeling “a bit lost” and “did not know what [they] had to give” whilst they were furloughed and that facilitating provided “some guidance” (Naomi).

Moreover, for some participants, the emotional connection came from in-the-moment facilitation which was described in terms of psychological ‘flow’ – a term put forward by Csikszentmihalyi and Csikszentmihalyi (1988) to describe a psychological state related to intrinsic motivation whereby individuals feel deeply immersed in an activity which they enjoy:

*“When you are facilitating sometimes, during that zone, you call it ‘in the flow’. You’re in that zone... and it’s only when you come out of it and somebody else has written their perception of what you deliver, that you have the time to reflect on what it was that you were talking about.” (Mina).*

*“*‘*Flow’ in this context is maybe about the kind of internal monologue of constant reflection and… going through a process together.” (Marie-anne).*

Thus, for these participants, their flow states when facilitating enabled a “reflective method” (Mina), whereby they were able to reflect on their own processes through others’ engagement with their facilitated dialogue. Further, participants felt that facilitating supported with feeling connected (“closer to the group,” Maya) and provided “a sense of belonging” (Mina), also making “people feel more invested” in the chats and the community.

Viewed together, these experiences align with McMillan and Chavis’ (1986, p. 9, pp. 11–12) theorization of ‘influence’ as important to creating a ‘sense of community,’ understood as a ‘sense of mattering.’ To feel a sense of belonging to a group, it is important to feel as though one is able to ‘make a difference’ to that group and to contribute in a way that means they ‘matter’ (McMillan and Chavis, 1986, p. 9). Through feeling a sense of purpose and having a valuable contribution to the group through facilitating, participants felt that they ‘mattered’. Moreover, this can be reinforced by ‘shared emotional connection’ (McMillan and Chavis, 1986, p. 9, 13–14), as we have seen here through shared flow and reflective states. This is interesting as it suggests the chats were able to find a balance between enabling freedom to choose how to engage and when to attend (aligning with earlier discussions of liminality), whilst also prompting participants to want to be integral to its processes and development (i.e., through facilitating) in a way that meant they ‘mattered’ and felt validated.

In sum of these themes, our chats fostered a sense of meaningful engagement and belonging to an online community. However, this community was not a ‘static’ community, but one that was characterized by processes and changing socioemotional experiences, aligning with the non-hierarchical conception of communitas. This communitas upholds a form of ‘networked individualism’ whereby participants could ‘be themselves’ as individuals, coming in and out of the space when they wished, and drawing upon both their personal and professional identities. The key mechanisms of this communitas were engaging in meaningful emotional experiences and having the opportunity to take on the role of being a ‘facilitator’ which fostered deeper connection to the community and enabled participants to derive psychosocial benefits from participation.

## Implications and conclusion

In this article, we have sought to explore whether it is possible to build a meaningful online community for those working in arts and play within the field of cultural and community engagement for health and wellbeing, and if such a community can support the psychosocial wellbeing of participants. Our findings highlight that constructing an online community with the core values of being inclusive and welcoming, whereby a consistent space is offered and active participation is at the heart of what it means to engage, can provide such psychosocial support. As our study took place during the COVID-19 pandemic, these findings reflect a particular moment in time where participants were able to engage in very few in-person interactions. This could also have had implications for our findings tending to be quite ‘positive,’ as participants were in a space in their lives where they were looking for social connection. If the study had been conducted later in the pandemic, the findings may have differed. Yet, although small in scale and conducted at just one time point, our study importantly shows that these processes provided consistent support and connection during the unstable time of the pandemic and supported with professional identity-building when the working lives of many of our participants were changing.

Online communities are not the same as in person, but they can offer something different that can be facilitative of meaningful emotional and social engagements. However, our research shows that an important part of creating such a community is offering a safe, supportive, non-hierarchical and creative space, whereby participants can choose how and when to engage. Those working within the community, health and cultural sectors, particularly those in freelance roles or on short-term contracts, need spaces for connection. There was a clear demand for spaces to connect and share experiences for those in these kind of roles during the pandemic and our online space began at a pivotal rupture in people’s lives (i.e., at the onset of the pandemic) and has acted as an important community hub to negotiate societal and personal changes *together*. The novel finding here is that this community provided support through allowing those within the community to set the direction of the chats (i.e., as facilitators) and to allow people to take the role within the community that they wanted, rather than taking a role within a pre-existing hierarchical structure. In our time of ‘networked individualism’ (Rainie and Wellman, 2012), this kind of meaningful online space is a new mode of shared engagement that can provide support against the backdrop of ever-increasing isolation, whereby participants can come and go when they choose. Although, it remains for future research to explore the longer-term sustainability of such a community and whether in our time of ‘permacrisis’ (Bendell, 2022), beyond COVID-19, online communities can *continue* to provide meaningful opportunities for connection.

This online community space is quite unique, as it is not tied to a particular research project or organization, and no prior knowledge is needed to engage. This unique online space holds semblance to community groups that exist in-person (e.g., run clubs, art drop in spaces, seniors’ craft and tea groups) where the environment, shared interest, and values welcome people and foster a relationship with the space. It is the ‘space’ and values connected to that space that are essential, and not having the same group of people each time. This is an interesting finding because it, in some ways, contradicts the very notion of an ‘online’ environment which cannot have a building that people can drop in and out of. Yet, the space fulfilled a similar space in people’s lives that offline community hubs can provide. As such, the online community group provided (and still provides) consistency and structure for people in an uncertain time. Rather than a building, the chats fostered a space that allows for meaningful digital co-presence where people are immersed and unable to multi-task for the hour. While this online community group formed in a particular time of extreme uncertainty, the study has implications for the potential of other online forums where participants can foster community across other times of crisis and uncertainty (ranging from pandemics, to natural disasters, to conflict, to mental health and addictions). Furthermore, the group itself is still flourishing at the time of writing (in 2023), even with other in-person options available and acts as both a consistent and new space for those engaged in art and play to connect.

Finally, although much literature shows the role of relationships with people to build communities (e.g., Jetten et al., 2011), our study highlights the role of relationships with the non-human world (i.e., the digital online space and to one’s own home environment, including material objects when drawn upon for creative engagement). One interesting area of future research would be to explore further whether the kind of people who engaged in our chats (e.g., those who had a connection to arts and play which often involved relations to art objects) may more readily be able to tap into the processes of a liminal communitas, when compared to those working in different careers, or whether the connective aspects of our online chats could have qualities that could provide support for those working in other precarious professional contexts.

## Data availability statement

The datasets in this article are not publicly available to preserve the anonymity of participants. To discuss this further, please contact KW via email on k.warran@ucl.ac.uk.

## Ethics statement

Ethical review and approval was not required for the study on human participants in accordance with the local legislation and institutional requirements. The patients/participants provided their written informed consent to participate in this study.

## Author contributions

KW and LW collaborated on the design and delivery of the study, independently analyzed the data and then synthesized findings across a series of meetings. All authors collaborated on the write-up of the manuscript and approved it for publication.

## Conflict of interest

The authors declare that the research was conducted in the absence of any commercial or financial relationships that could be construed as a potential conflict of interest.

## Publisher’s note

All claims expressed in this article are solely those of the authors and do not necessarily represent those of their affiliated organizations, or those of the publisher, the editors and the reviewers. Any product that may be evaluated in this article, or claim that may be made by its manufacturer, is not guaranteed or endorsed by the publisher.
