# Supplementary material for: Online ‘chats’: fostering communitas and psychosocial support for people working across arts and play for health and wellbeing
Source: Front Psychol. 2023 Jul 24;14:1198635. doi: 10.3389/fpsyg.2023.1198635 (PMC10405829; doi:10.3389/fpsyg.2023.1198635)
Supplement: Supplementary file 1 [file Presentation_1.pdf]

## Supplementary file

Interview guide

**Introduction**

*Welcome everyone to the focus group Discussion. Provide an overview of the purpose of the focus group discussion, its duration (90 minutes), structure (arts based and participatory), anonymity, and consent (go over form and see if anyone has questions, ask if comfortable recording).*

Please say your name and if you feel comfortable a word/sentence about a moment where you felt a sense of connection with others or your environment.

**Activity:** River Journey

**Purpose:** To explore participants feelings and experience of social cohesion over the journey of the Social Isolation to Social Cohesion chats (March to Present)

**Materials**: paper, pens/markers/pastels

**Process:**

1. Ask participants to close their eyes and envision a beautiful river, flowing from the start to the present and into the future.
2. Ask participants to reflect on how they were feeling when they first joined the chats, in the middle, and what they feel for the future. What are some of the most significant experiences and/or changes in relation to social cohesion (personally, professionally, in community)?
3. Invite participants to take out their piece of paper and colours. Ask participants to draw a long windy river across their page. Ask them to write start on the left side, middle in the centre, and present/future on the right.
4. Ask participants to take five to ten minutes to draw or write their stories and ideas.

**Semi Structured Questions**

1. Would anyone like to start by sharing their drawing and saying a little bit about why you decided to engage with the online chats? How did you hear about them and why did you decide to join?
2. How would you describe your experiences of engaging with the online chats? *Prompts: what words come to mind?*
3. How has attending the chats made you feel? *Prompts: did you feel connected? Reflective? What other feelings come to mind?*
4. How did you find engaging in a group setting? *Prompts: what supported you to engage? What reduced your level of engagement? Did you feel that you 'belonged' to the group or would you use another word? Was it important that the sessions were structured as group discussions? If yes, why? If no, why not?*
5. Was there anything discussed in the sessions which influenced action outside of them? For example, did you take action in relation to implementing resources that were discussed or in changing program activities based on discussions? *Prompts: Have you connected with anyone from the chats independently on a personal or professional level? If so, what are some of the types of connections and actions you have made? Are there other types of connections or areas of social cohesion you would like to explore in the chats going forward or outside of this group?*
6. What role have the online chats played for you personally in light of the context of the Covid-19 pandemic? What, if any, are "golden nuggets" you intend to take forward from your engagement with the online chats? *Prompts: do you think the experience of engaging online*

*has been different because of lockdown? How? Would engaging in an online chat prior to lockdown have played the same role?*

7. All of you have facilitated or co-facilitated a session for the social cohesion chats. How did facilitating effect your feelings of social cohesion and connection to the group?
8. Is there anything else you would like to share you feel is relevant to the study?
9. Do you have any questions for us?
